# Supplementary material for: Flexible three-dimensional artificial synapse networks with correlated learning and trainable memory capability
Source: Nat Commun. 2017 Sep 29;8:752. doi: 10.1038/s41467-017-00803-1 (PMC5622032; doi:10.1038/s41467-017-00803-1)
Supplement: Supplementary file 1 — Supplementary Information [file 41467_2017_803_MOESM1_ESM.pdf]

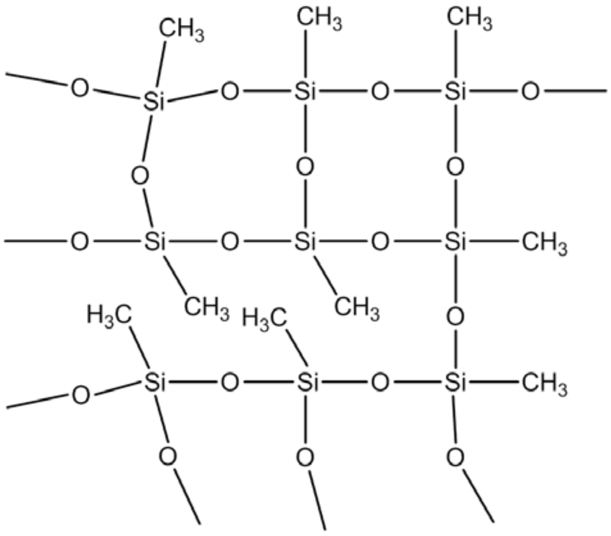

Supplementary Figure 1. Schematic structure of pMSSQ.

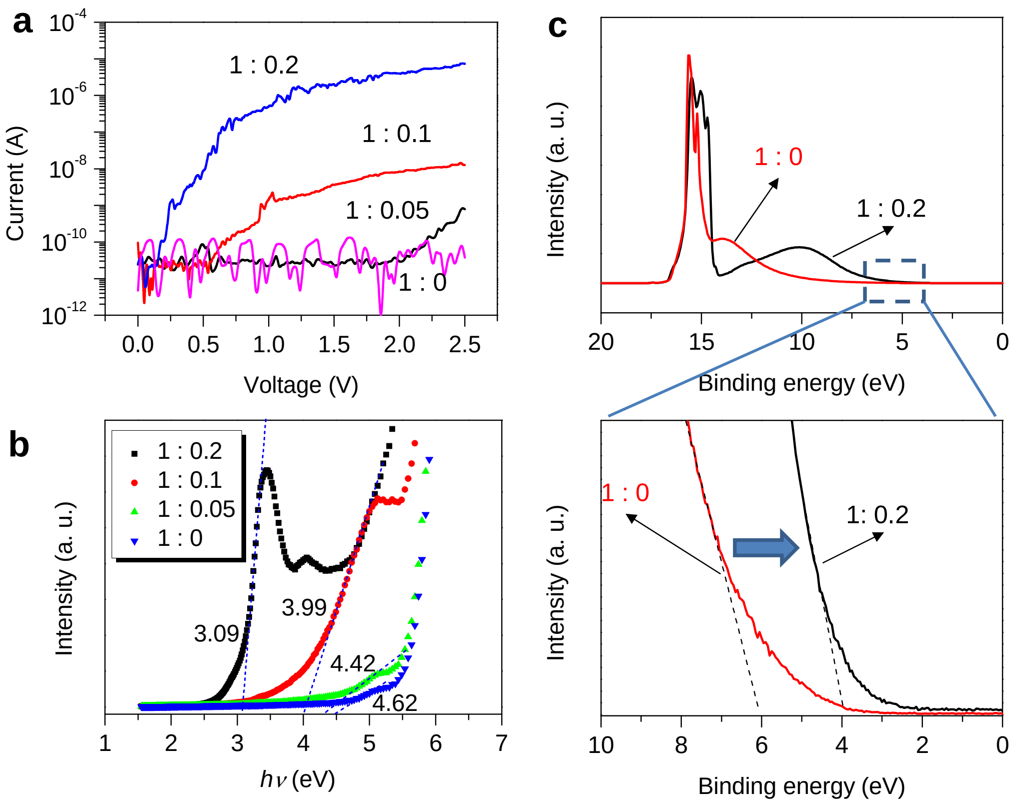

Supplementary Figure 2. Electric properties of Cu-doped pMSSQ. (a) Current-voltage curves of samples with a structure of Al/Cu-doped pMSSQ/Al. (b) Absorption coefficients for samples with different Cu ion concentrations. (c) UPS spectra of pure pMSSQ and Cu-doped pMSSQ. The bottom plane shows the spectral region around the HOMO of pure pMSSQ and Cu-doped pMSSQ presented. For the preparation of the samples, the methyltrimethoxysilane precursor and the $CuCl_2$ aqueous solution are mixed in volume ratios of 1:0.2, 1:0.1, 1:0.05, and 1:0.

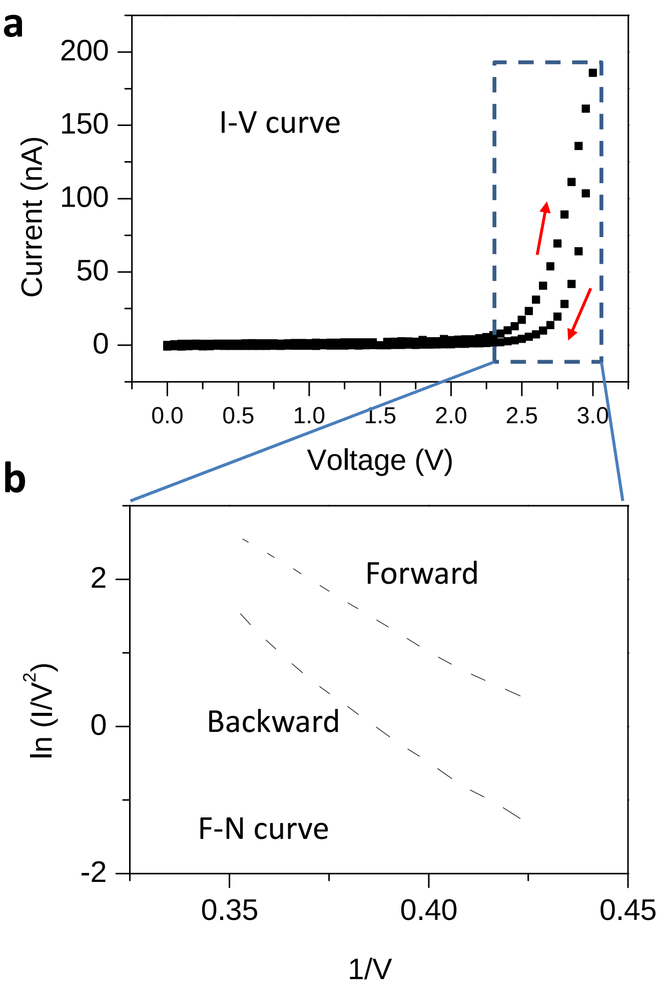

Supplementary Figure 3. (**a**) *I-V* curves of an e-synapse under positive bias. (**b**) The related Fowler-Nordheim (*F-N*) curves. The linear relation between $1/V$ and $\ln(I/V^2)$ indicates *F-N* tunneling. The difference in the slopes of the $\ln(I/V^2)$ versus $1/V$ curves for the forward and the backward applied voltages indicates variations in the characteristics of the energy barriers. The larger slope for the backward sweep indicates that the energy barrier is higher.

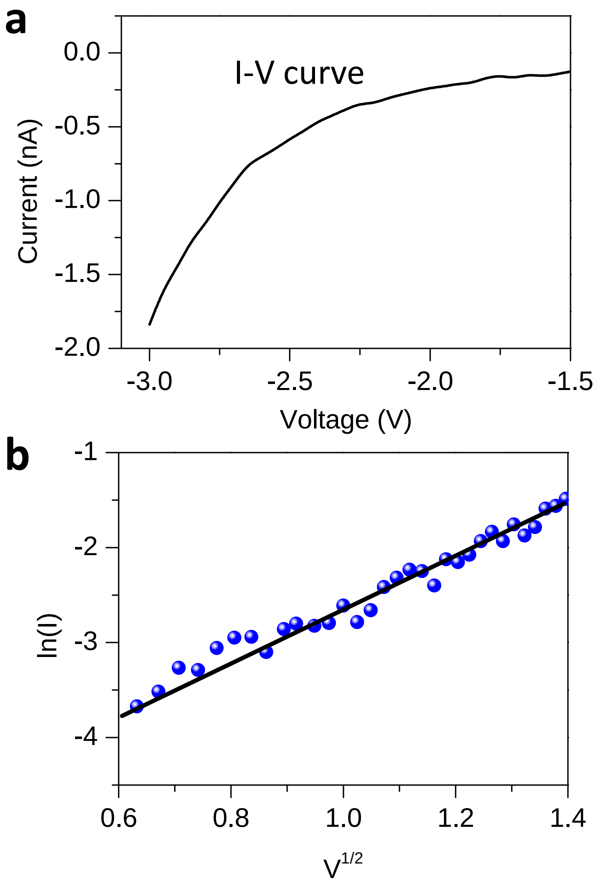

Supplementary Figure 4. (**a**) *I-V* curve of an e-synapse under negative bias. (**b**) ln(*I*) versus $V^{1/2}$ fitted curve. The linearity indicates that thermionic emission (TE) dominates carrier transport in the device in the low-voltage region.

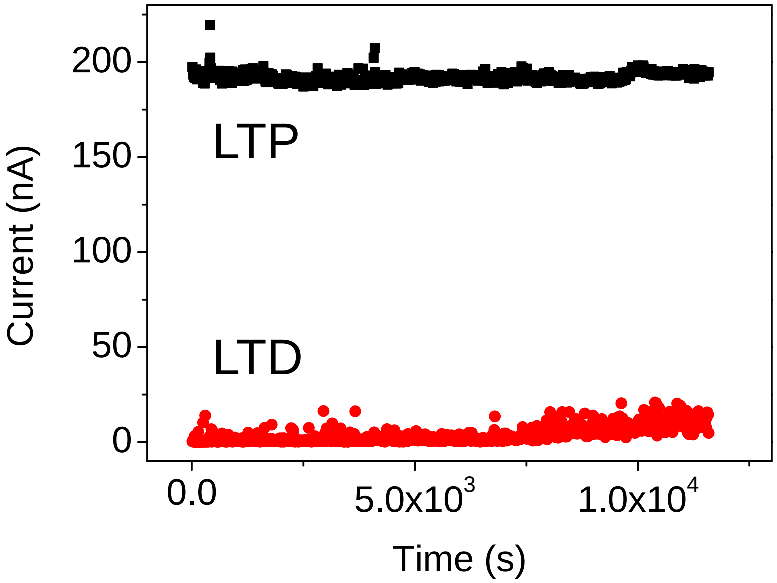

Supplementary Figure 5. Long-term stability of e-synapse. A series of 3-V (-3-V) pulses were applied to achieve low (high) conductance states. No discernible degradation in the readout current was observed during the test time of 10^4 s. The excellent retention results demonstrate that the e-synapse can mimic simultaneously the long-term potentiation (LTP) and the long-term depression (LTD) properties of a biological synapse.

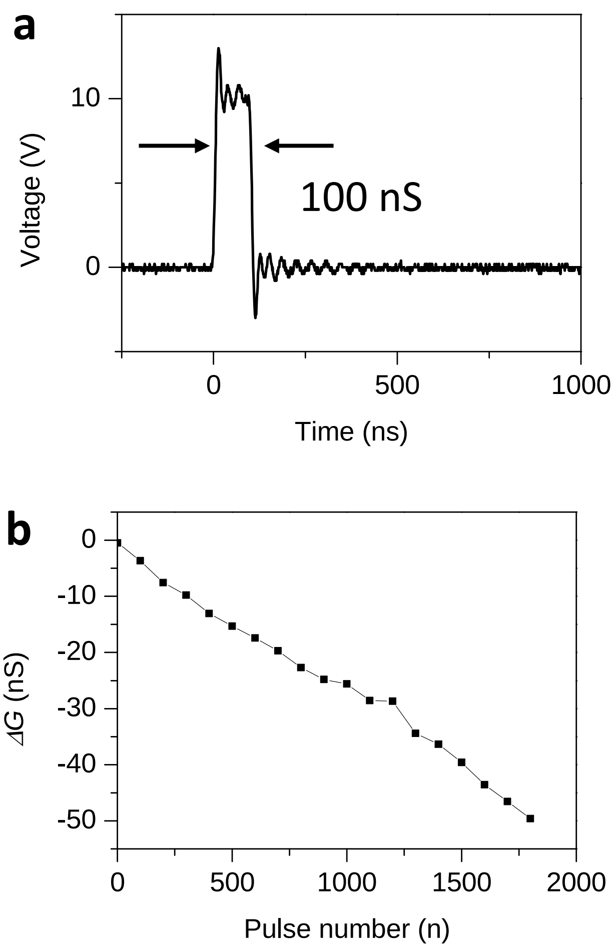

Supplementary Figure 6. (a) Waveform of the positive pulse (10 V, 100 ns) applied to the e-synapse. (b) ΔG of the e-synapse as a function of the number of the positive pulses.

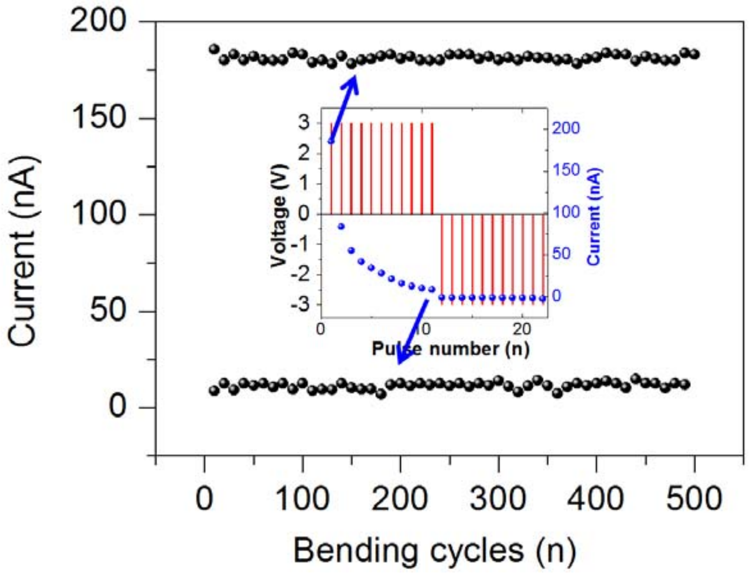

Supplementary Figure 7. Electrical stability of 3D-ASN under 500 mechanical bending cycles. In this measurement, the sample was bent with a radius of curvature of 10 mm for 500 cycles. Each bending cycle included one compression and one extension of the functional film. After every 10 bending cycles, positive and negative pulses (inset of Supplementary Fig. 7) were applied to measure the electrical properties of the e-synapse. The currents in the e-synapse at the 1st positive pulse and the 11th positive pulse were recorded as the low- and the high-resistance states, respectively. This result shows that the 3D-ASN exhibits outstanding mechanical deformation endurance.

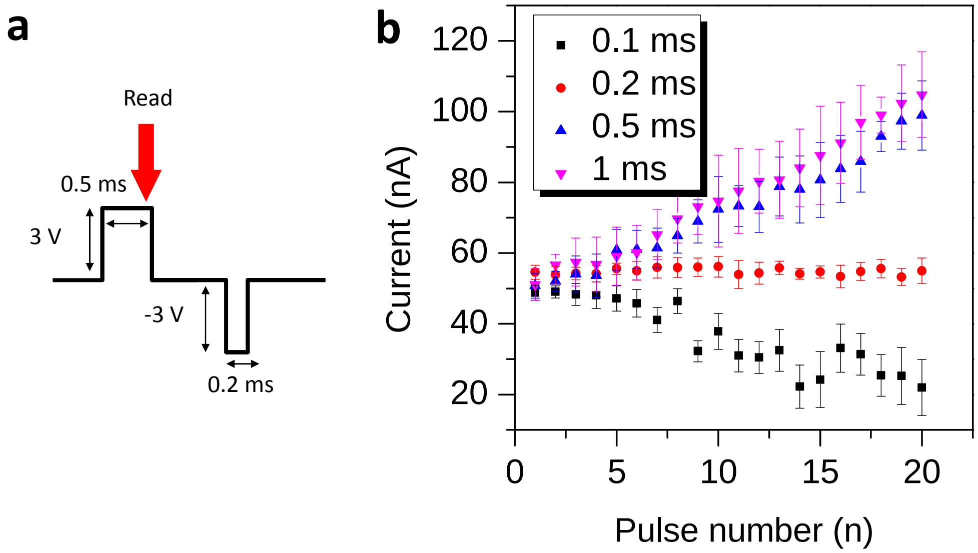

Supplementary Figure 8. (**a**) Waveform of pulse trains for the reading process. (**b**) Variation in the readout current when using different reading pulse trains. Note that a large positive reading voltage pulse might decrease the readout current significantly. As a result, the reading process of the e-synapse with a positive bias pulse is destructive. We try to avoid the destructive readout process by applying the 3-V reading pulse first and subsequently applying the -3-V pulse to achieve the conduction state. Each pulse train consists of a reading pulse (3 V, 0.5 ms), followed by a negative pulse (-3 V), as shown in Supplementary Fig. 8a. The readout currents as functions of the number of pulses for different pulse widths for the -3-V pulses are shown in Supplementary Fig. 8b. While the readout currents for a wider -3-V pulse gradually increase, those for a narrower -3-V pulse gradually decrease. When the width of the -3-V pulse is 0.2 ms, the readout current is almost constant, indicative of a nondestructive reading process.

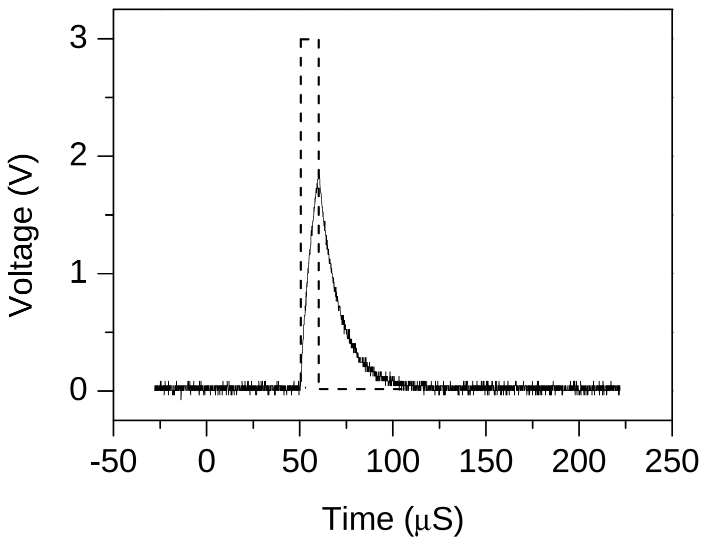

Supplementary Figure 9. Recorded waveform of the pulse applied to the e-synapse. The real spike (solid lines, 3 V, 10 μs) applied to the e-synapse is different from the square pulse (dash lines) generated from the signal generator due to a charge-discharge process in the resistor-capacitor loop. The real peak voltage applied to the e-synapse is 1.8 V.
